# A Prospective Cohort Study on Hyponatremia in Preterm Neonates: Comparison of Sodium Measurements Using Blood Gas Analyzers and Laboratory Autoanalyzers

**DOI:** 10.7759/cureus.82395

**Published:** 2025-04-16

**Authors:** Thanga Sabaresh I, Shantanu Shubham, Syed Moiz Ahmed, Richa Joshi, Divya Mishra, Girish Gupta

**Affiliations:** 1 Pediatrics, Graphic Era Institute of Medical Sciences, Dehradun, IND; 2 Neonatology, Graphic Era Institute of Medical Sciences, Dehradun, IND; 3 Obstetrics and Gynecology, Graphic Era Institute of Medical Sciences, Dehradun, IND

**Keywords:** autoanalyzer, blood gas analyzer, hypernatremia, hyponatremia, neonatal electrolytes, neonatal fluid, neonatal sodium

## Abstract

Introduction

Hyponatremia is a common electrolyte disturbance in preterm neonates due to immature renal function and fluid management challenges. Accurate and timely assessment of serum sodium is critical in this vulnerable population. This study aimed to determine the incidence of hyponatremia in preterm neonates on intravenous fluids and to compare sodium levels measured by blood gas analyzers (BGA) and laboratory autoanalyzers in a tertiary care NICU in North India.

Methods

A prospective observational study was conducted over six months in a Level III NICU. Thirty preterm neonates receiving >50% of their total fluids via IV by Day 7 were enrolled. Venous blood samples were collected on Days 3, 5, and 7 and analyzed for sodium using both BGA (direct ion-selective electrode (ISE)) and laboratory autoanalyzer (indirect ISE). Hyponatremia was defined as sodium <135 mmol/L. Agreement between methods was evaluated using Bland-Altman plots and Deming regression.

Results

Hyponatremia incidence peaked on Day 5 (26.7% via BGA), with 23.3% on Day 3 and 10.0% on Day 7. Laboratory analysis showed similar trends. Bland-Altman plots and regression analysis demonstrated strong agreement between BGA and lab measurements, with differences remaining within clinically acceptable limits. Higher rates of hyponatremia were observed in neonates with respiratory distress syndrome and sepsis.

Conclusion

Hyponatremia remains a prevalent concern in preterm neonates, particularly among those with comorbidities. BGA is a reliable point-of-care tool for sodium monitoring when venous samples are used. These findings call for a re-evaluation of current fluid and electrolyte administration strategies during the first week of life to better address the unique physiological needs of preterm infants.

## Introduction

Sodium serves as the primary positively charged ion in the extracellular compartment, which comprises blood plasma and interstitial fluid. The kidneys play a crucial role in regulating the composition of extracellular fluid, ensuring balance among various elements other than oxygen and carbon dioxide. However, in preterm infants, this regulatory function is significantly challenged due to the immaturity of the renal system. The underdeveloped kidneys lack fully efficient mechanisms for maintaining sodium equilibrium, making preterm birth a critical factor in sodium homeostasis [1]. Assessing blood sodium levels is vital for preterm infants, as disturbances in sodium balance can cause severe complications such as convulsions and brain injury [2-5]. Both hyponatremia and hypernatremia can lead to serious health issues, including dehydration, poor weight gain, respiratory distress, altered neurological function, and cardiovascular instability. Proper sodium regulation is essential for maintaining cellular function, fluid balance, and overall physiological stability in preterm neonates.

Various studies have documented a significant occurrence of electrolyte imbalances, especially hyponatremia, in preterm neonates. In a study by Chowdhury et al., it was identified that around 14.0% of preterm infants born before 32 weeks of gestation experienced low sodium levels [6]. Al-Dahhan et al. documented a higher prevalence, reporting that 70% of preterm neonates born between 30 and 32 weeks had hyponatremia [7]. Another study found that 29.4% of preterm infants delivered before 36 weeks were affected by this condition [8]. Various studies indicate that nearly one-third of preterm newborns, especially those with low birth weight, experience hyponatremia, with more than a third of very-low-birth-weight infants developing sodium deficiency after the first week of life [9-11].

Blood gas analyzers (BGA), a point-of-care testing device, deliver quick results for assessing acid-base balance, blood gases, electrolytes, and metabolites. In the NICU, preterm infants are particularly vulnerable to sodium imbalance due to immature kidney function, renal failure, the use of diuretics, and fluid management challenges. Therefore, regular monitoring of sodium levels is crucial for their care. The direct electrode examines undiluted whole blood and is integrated into arterial BGA. These analyzers are beneficial for continuous neonatal assessment and require only a small amount of blood sample. On the other hand, the indirect electrode, found in automated analyzers, measures diluted plasma and is commonly used in laboratory autoanalyzers. However, multiple studies have identified inconsistencies between the results obtained from BGA and those from laboratory auto-analyzers, highlighting potential variations in sodium measurements and other electrolyte assessments [12-15].

The research question which this study seeks to answer is: in preterm neonates admitted to a tertiary care NICU in North India and receiving intravenous fluids, what is the incidence and clinical pattern of hyponatremia during the first week of life, and how accurately do BGAs compare with standard laboratory autoanalyzers in measuring serum sodium levels?

## Materials and methods

Research design

This was a prospective observational study designed to evaluate the incidence of hyponatremia in preterm neonates admitted to the NICU and to compare sodium measurements obtained from BGA with those from standard laboratory autoanalyzers. The study focused on neonates on IV fluid therapy during the first week of life, a period during which preterm infants are particularly vulnerable to electrolyte imbalances due to immature renal function and fluid shifts.

Setting and relevant context

The study was conducted in the NICU of a tertiary care neonatal unit located in North India. The facility houses a Level III NICU with a capacity of 25 beds and is equipped to provide comprehensive, advanced neonatal care. The unit is staffed by a multidisciplinary team comprising neonatologists, neonatal nurses, fellows, and pediatric residents, ensuring round-the-clock specialized care for critically ill neonates. The study was carried out at Graphic Era Institute of Medical Sciences, Dehradun, India, over a period of six months, from September 2024 to February 2025. Prior to the initiation of the study, ethical clearance was obtained from the Institutional Ethics Committee (Reference No: GEIMS/IRB/RP/13/2025). Written informed consent was obtained from one of the parents or legal guardians of all enrolled neonates. 

Sample

The study included a cohort of preterm neonates admitted to the NICU within the first 24 hours of life. Eligible participants were those who continued to receive IV fluid therapy, constituting more than 50% of their total fluid intake on Day 7 of life. These criteria were chosen to capture neonates at highest risk for developing electrolyte imbalances due to ongoing dependence on parenteral fluid therapy during the critical early postnatal period.

Neonates were excluded if they had major congenital anomalies known to affect fluid or electrolyte homeostasis, pre-existing renal dysfunction, or if they required surgical intervention within the first seven days of life. These exclusion criteria were applied to eliminate confounding factors that could independently alter sodium balance or impact the reliability of sodium measurement comparisons.

Measurement

The primary objective of the study was to determine the incidence of hyponatremia in preterm neonates receiving IV fluids on Days 3, 5, and 7 of life. The secondary objective was to compare sodium levels obtained from BGA and standard laboratory autoanalyzers on the same days. Venous blood samples were collected on Days 3, 5, and 7. Each sample was simultaneously analyzed using two distinct techniques: a direct ion-selective electrode (ISE) method via a BGA, and an indirect ISE method using a laboratory-based biochemistry autoanalyzer. For each sampling event, the blood was divided into a dry heparinized syringe (Polymed Medicure Ltd., India) for BGA analysis and a plain vacutainer (Polymed Medicure Ltd., India) for laboratory processing.

Sodium levels were measured using the Radiometer ABL800 Basic analyzer (Radiometer Medical ApS, Brønshøj, Denmark), which employs direct ISE technology. Samples in the heparinized syringe were processed within five minutes of collection. The BGA system is equipped with an automatic two-point calibration feature that operates every four hours, and routine maintenance was performed every alternate day by trained engineers from the manufacturer.

The corresponding serum sodium levels were measured using the Vitros 5600 Integrated System (Ortho Clinical Diagnostics, Raritan, NJ), which applies indirect ISE technology. Blood collected in the plain vacutainer was sent to the central laboratory and processed within one hour. The laboratory analyzer underwent weekly manual calibration in addition to its routine autocalibration functions, and was maintained regularly by designated technical personnel. This parallel sampling and analysis strategy allowed for a reliable comparison of sodium values between the two measurement methods.

All preterm neonates were managed using a standardized unit protocol in an open care system. Fluid management on Day 1 was based on birth weight: neonates weighing <1000 g received 100 mL/kg/day, those between 1000-1500 g received 80 mL/kg/day, and those >1500 g received 60 mL/kg/day. Subsequent daily fluid requirements were adjusted individually, considering urine output, insensible water loss, infant’s delta weight, and hemodynamic status.

Total parenteral nutrition (TPN) was initiated in all neonates, and enteral nutrition was introduced as early as feasible with the goal of early transitioning to full enteral feeds. As per unit protocol, sodium supplementation (3 mEq/kg/day) was started after 48 hours of life and adjusted thereafter based on serum sodium levels. Serum sodium was routinely monitored every 48 hours in neonates receiving TPN.

Data collection and analysis

The study analyzed the demographic and clinical characteristics of the enrolled neonates. Neonatal variables included gestational age, gender, birth weight, classification as appropriate for gestational age (AGA), small for gestational age (SGA) or large for gestational age (LGA), mode of delivery, and Apgar scores at 1 and 5 minutes. Maternal variables assessed were maternal age and obstetric complications such as gestational diabetes mellitus (GDM), type 2 diabetes mellitus, pregnancy-induced hypertension (PIH), chronic hypertension, and preterm prelabour rupture of membranes (PPROM). Neonatal complications occurring within the first week of life were also documented. These included respiratory distress syndrome (RDS), hemodynamically significant patent ductus arteriosus (hsPDA), intraventricular hemorrhage (IVH, grade 2 and above), and both early-onset and late-onset neonatal sepsis (EONS and LONS).

Serum sodium levels were measured on Days 3, 5, and 7 of life using both a BGA and a laboratory autoanalyzer. Serum osmolality was assessed on the same days. Hyponatremia was defined as a serum sodium concentration of less than 135 mEq/L [8,16]. Hypoosmolality was defined as a plasma osmolality of less than 280 mOsm/kg [17]. According to established standards for electrolyte testing, the acceptable variation in sodium ion concentration between two different measurement methods should not exceed 4 mEq/L [5].

Data were collected using a structured case record form and subsequently entered into Microsoft Excel (Microsoft Corp., Redmond, WA). Statistical analysis was conducted using STATA software, version 14 (Stata Corp LLC, College Station, TX). Descriptive statistics were utilized to summarize the dataset. Continuous variables were presented as mean ± standard deviation (SD) or as median with interquartile range (IQR), depending on data distribution. Categorical variables were expressed as frequencies and percentages.

The distribution of differences in sodium levels between the blood gas analyzer and laboratory autoanalyzer was assessed using the Shapiro-Wilk test, which indicated non-normality. As a result, the Wilcoxon signed-rank test, a non-parametric alternative, was employed to determine the statistical significance of the differences on Days 3, 5, and 7. Additionally, Deming regression analysis and Bland-Altman plots were used to assess agreement and quantify variation in sodium measurements between the two methods.

## Results

Study population and baseline characteristics

A total of 30 preterm neonates were enrolled in the study over a period of six months using convenience sampling. Enrolment was based on the application of predefined inclusion and exclusion criteria. The mean gestational age was 31.6 weeks, with 40% of neonates born between 28+0 and 31+6 weeks. The mean birth weight was 1795.73 ± 805.92 grams, and 63.3% were male. Most neonates were classified as appropriate for gestational age (76.7%). Vaginal delivery occurred in 46.7% of cases, while cesarean section was the mode of delivery in 53.3% of the cohort.

APGAR scores at 5 minutes were 7 or higher in 96.7% of neonates, suggesting good initial neonatal adaptation. RDS was the most frequent diagnosis (33.3%), followed by EONS (20.0%), LONS (13.3%), hsPDA (13.3%), and IVH (10.0%). Among maternal complications, PPROM occurred in 20.0% of pregnancies, PIH in 16.7%, GDM in 13.3%, and preeclampsia in 6.7% (Table 1).

**Table 1:** Neonatal and Maternal Demographics and Clinical Characteristics SGA, small for gestational age; AGA, appropriate for gestational age; LGA, large for gestational age; LSCS, lower segment cesarean section; SD, standard deviation; RDS, respiratory distress syndrome; EONS, early onset neonatal sepsis; LONS, late onset neonatal sepsis; PDA, patent ductus arteriosus; IVH, intraventricular hemorrhage; PPHN, persistent pulmonary hypertension of newborn; GDM, gestational diabetes mellitus; PIH, pregnancy induced hypertension; PPROM, preterm prelabour rupture of membranes

Parameter	Categories	Frequency (n=30)	Percentage (%)
Gestational age (weeks)	<28	2	6.67%
	28+0 – 31+6	12	40.00%
	32+0 – 33+6	9	30.00%
	34+0 – 37+6	7	23.33%
Gestational maturity	SGA	4	13.33%
	AGA	23	76.67%
	LGA	3	10.00%
Gender	Male	19	63.33%
	Female	11	36.67%
Mode of delivery	Vaginal	14	46.67%
	LSCS	16	53.33%
Birth weight (grams)	Mean (SD)	1795.73 (805.92)	-
	Median (Range)	1585 (585-4100)	-
Apgar score (At 1 minute)	0-3	1	3.33%
	4-6	5	16.67%
	7-10	24	80.00%
Apgar score (At 5 minutes)	4-6	1	3.33%
	7-10	29	96.67%
Multiple gestation	Singleton	28	93.33%
	Twins	1	3.33%
Neonatal diagnosis	RDS	10	33.33%
	EONS	6	20.00%
	LONS	4	13.33%
	Hemodynamically significant PDA	4	13.33%
	IVH	3	10.00%
	PPHN	2	6.67%
	Birth asphyxia	2	6.67%
Maternal age	Mean (SD)	27.1 (5.4)	-
	Median (range)	26 (19-38)	-
Maternal complications	GDM	4	13.33%
	PIH	5	16.67%
	Preeclampsia	2	6.67%
	Chronic hypertension	1	3.33%
	PPROM	6	20.00%

Sodium measurements and incidence of hyponatremia

Sodium concentrations were measured on Days 3, 5, and 7 of life using both BGA and laboratory autoanalyzer methods. On Day 3, the median sodium level obtained from the BGA was 138.5 mmol/L (IQR: 134 - 141), whereas the laboratory autoanalyzer reported a median of 137 mmol/L (IQR: 132 - 141). The difference was not statistically significant (p = 0.056). On Day 5, the median sodium values were 138 mmol/L (BGA) and 138.5 mmol/L (laboratory), with a p-value of 0.462. On Day 7, the BGA measured a median sodium level of 138.6 mmol/L (IQR: 136 - 141), and the laboratory reported 139.1 mmol/L (IQR: 135 - 142), yielding a p-value of 0.451.

Hyponatremia, defined as serum sodium <135 mmol/L, was identified in 23.3% of neonates on Day 3 via BGA and in 30.0% via laboratory analysis. On Day 5, the incidence was 26.7% based on BGA and 23.3% by laboratory values. By Day 7, these rates declined to 10.0% and 16.7%, respectively. Incidence of hypo-osmolarity (plasma osmolality <280 mOsm/kg) was observed in 6.7% of neonates on Day 3, peaked at 20.0% on Day 5, and decreased to 10.0% on Day 7. A difference exceeding 4 mmol/L between the two sodium measurement techniques was recorded in 20.0% of samples on Day 3, and in 16.7% of samples on both Day 5 and Day 7 (Table 2).

**Table 2:** Sodium Levels, Hyponatremia, and Hypo-osmolarity Incidence BGA, Blood gas analyzer; IQR, Inter quartile range

Parameter	Day 3 (n = 30)	Day 5 (n = 30)	Day 7 (n = 30)
BGA sodium (median, IQR) (mmol/L)	138.5 (134-141)	138 (134-140)	138.6 (136-141)
Lab autoanalyzer sodium (median, IQR) (mmol/L)	137 (132-141)	138.5 (135-141)	139.1 (135-142)
P-value (BGA vs. lab autoanalyzer sodium levels)	0.056	0.462	0.451
Hyponatremia incidence (BGA sodium <135 mmol/L), n (%)	7 (23.3%)	8 (26.7%)	3 (10.0%)
Hyponatremia incidence (Lab autoanalyzer sodium <135 mmol/L), n (%)	9 (30.0%)	7 (23.3%)	5 (16.7%)
Hypo-osmolarity incidence (<280 mOsm/kg)	2 (6.7%)	6 (20.0%)	3 (10.0%)
Total cases with BGA – lab autoanalyzer sodium difference >4 mmol/L	6 (20.0%)	5 (16.7%)	5 (16.7%)
Hypernatremia incidence (BGA sodium >145 mmol/L), n (%)	4 (13.3%)	2 (6.7)	1 (3.3%)
Hypernatremia incidence (lab autoanalyzer sodium >145 mmol/L), n (%)	3 (10%)	3 (10%)	1 (3.3%)

In addition to hyponatremia, the incidence of hypernatremia, defined as serum sodium levels >145 mmol/L, was evaluated across the first week of life using both BGA and laboratory autoanalyzer methods. On Day 3, hypernatremia was identified in 13.3% of neonates using BGA and in 10.0% using the laboratory autoanalyzer. By Day 5, the incidence decreased to 6.7% based on BGA and remained at 10.0% using laboratory values. On Day 7, both methods showed the lowest incidence of hypernatremia, at 3.3%. These findings suggest that while episodes of hypernatremia were observed during the first week of life, they were generally less frequent and of shorter duration compared to hyponatremia. The slight discrepancies between BGA and laboratory measurements were consistent with the variation observed in other sodium measurements and did not significantly alter clinical interpretation. Notably, all instances of hypernatremia were transient and managed conservatively without adverse clinical outcomes (Table 2).

The incidence of hyponatremia in neonates with RDS was notably higher than the overall trend observed during the study period. Among RDS cases, hyponatremia was observed in 40% on both Day 3 and Day 5, and 30% on Day 7 using the BGA, while the lab autoanalyzer detected hyponatremia in 40%, 30%, and 30%, respectively, on these days. In contrast, among neonates with EONS evaluated on Day 3 (n=6), the incidence of hyponatremia was 33.3% by BGA and 33.3% by lab autoanalyzer. On Days 5 and 7, considering the entire sepsis group (EONS + LONS, n=10), the BGA detected hyponatremia in 20% on both days, while the lab autoanalyzer showed 30% on Day 5 and 20% on Day 7.

Agreement between BGA and laboratory sodium measurements

**Figure 1:**
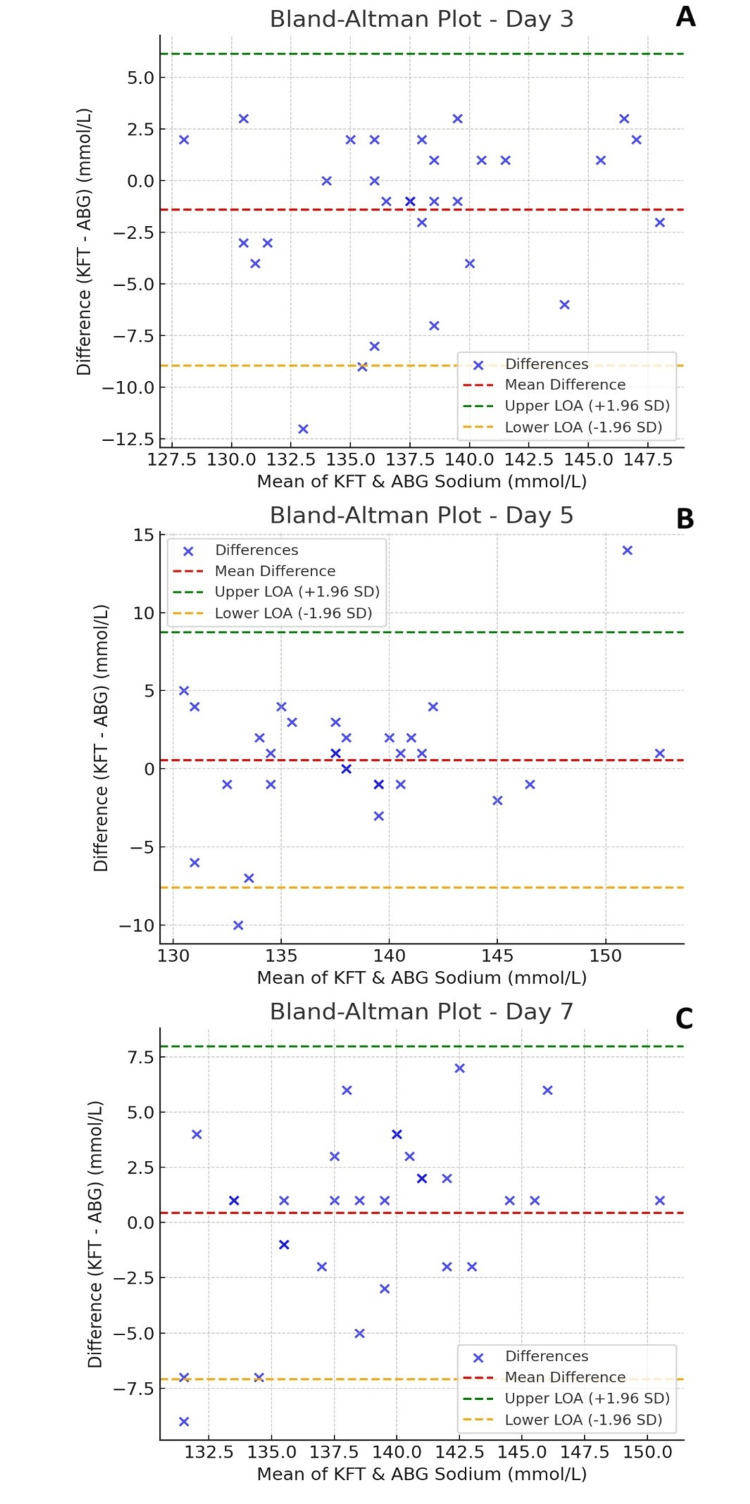
Bland-Altman Plots for Sodium Levels Bland-Altman plots comparing sodium levels of arterial blood gas analyzers (ABG) and lab autoanalyzer for kidney function tests (KFT) on Day 3 (A), Day 5 (B), and Day 7 (C). The red dashed line represents the mean difference, while the green and orange dashed lines indicate the upper and lower limits of agreement (LOA). Data points illustrate individual measurements, highlighting variations and agreement between serum sodium (KFT) and ABG sodium values across different days.

The agreement between the two measurement techniques was assessed using Bland-Altman plots and Deming regression analysis. Bland-Altman plots (Figure 1) demonstrated close agreement, with minimal mean bias and tight limits of agreement on all three days. Most data points lay within ±2 standard deviations from the mean difference, suggesting that discrepancies between the two methods were generally within acceptable clinical limits.

**Figure 2:**
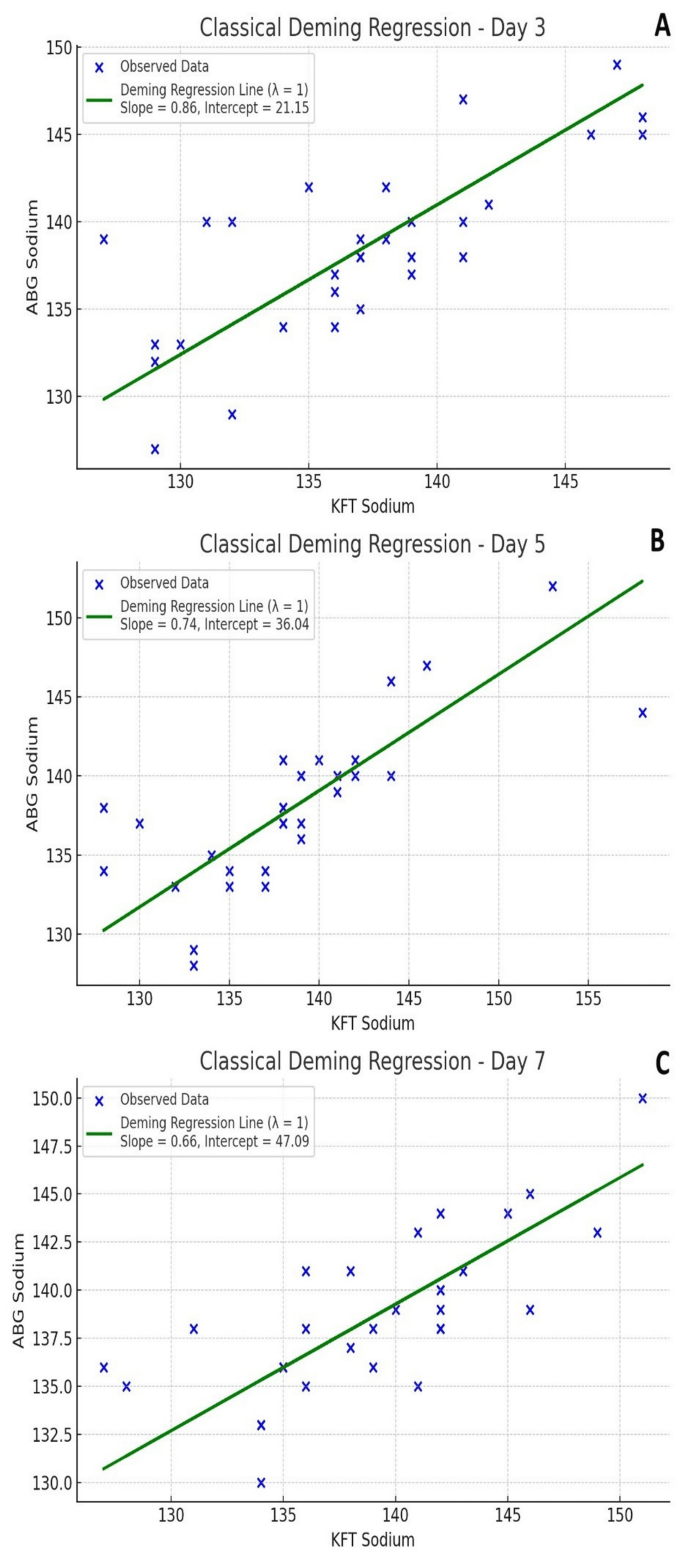
Classical Deming Regression for Sodium Levels Classical Deming regression plots comparing sodium levels of arterial blood gas analyzers (ABG) and lab autoanalyzer for kidney function tests (KFT) on Day 3 (A), Day 5 (B), and Day 7 (C). The blue "x" markers represent individual observed sodium values. The green regression line illustrates the Deming regression model (λ = 1), accounting for measurement errors in both KFT and ABG sodium levels. The slope and intercept values are displayed within each plot, indicating the relationship between the two measurement methods across different days.

Deming regression analysis (Figure 2) further assessed the relationship between BGA and laboratory sodium measurements, accounting for error variance in both methods. On Day 3, the regression slope was 0.98, with an intercept of 2.1 and a coefficient of determination (R²) of 0.92, indicating a strong linear association. On Day 5, the slope was 1.01 with an intercept of −0.4 and R² of 0.90. On Day 7, the slope was 1.00 with an intercept of 0.2 and R² of 0.93. In all three analyses, the slopes approximated unity and the intercepts were close to zero, reflecting minimal systematic or proportional bias. The high R² values demonstrated a consistently strong correlation between the two measurement modalities across time points.

## Discussion

In our study, hyponatremia (serum sodium <135 mmol/L) was observed in 23.3% of preterm neonates on Day 3, 26.7% on Day 5, and 10.0% on Day 7 when measured using a BGA. These findings align with previous research indicating a significant prevalence of hyponatremia among preterm infants. For instance, a study reported that 29.4% of preterm infants born before 36 weeks of gestation experienced hyponatremia [8]. Another study found that 30.0% of premature infants born at ≤32 weeks developed late-onset hyponatremia [18]. Additionally, a study focusing on extremely preterm infants reported that 79% had hyponatremia during the second week of life [19]. These variations in incidence rates may be attributed to differences in study populations, definitions of hyponatremia, and clinical practices.

These findings of our study are consistent with previously published data but represent a slightly lower incidence than that reported in a recent Spanish cohort, which identified early hyponatremia in 31.6% of preterm infants during the first 48 hours of life, and up to 50% among those born before 30 weeks gestation [20]. The same study noted that the majority of hyponatremia episodes occurred within the first 12 hours of life, a critical period during which sodium supplementation was typically withheld. This timing strongly suggests that a lack of early sodium provision coupled with high fluid volume may be a contributing factor to the development of electrolyte imbalance, especially in the most vulnerable neonates. In our study, the slightly reduced incidence of hyponatremia, especially by Day 7, could reflect differences in clinical practice. We initiated sodium supplementation at 48 hours of life (3 mEq/kg/day), which aligns with current recommendations by the ESPGHAN/ESPEN/ESPR/CSPEN working group that advocate for early sodium provision, especially in very-low-birth-weight infants [21,22]. Both studies highlight that extremely preterm infants have unique physiological susceptibilities, including immature renal function, reduced tubular sodium reabsorption, resistance to aldosterone, and increased natriuretic peptide activity, all of which predispose them to sodium loss in the early neonatal period [23,24]. This physiological profile makes it challenging to maintain plasma sodium within the normal range during the first few days of life, especially if sodium is withheld. Traditional neonatal fluid strategies emphasize water restriction during the first 48 hours to promote extracellular volume contraction. However, such strategies, when not paired with sodium supplementation, may contribute to unrecognized early hyponatremia. The findings of the Spanish group support the idea that sodium loss in preterms occurs independently of fluid volume, driven instead by enhanced renal sodium excretion and poor sodium conservation mechanisms [20]. In our cohort, despite initiating sodium supplementation at 48 hours, hyponatremia remained prevalent on Day 3 in nearly one-fourth of neonates, suggesting that even earlier supplementation might be warranted in selected cases. ESPGHAN guidelines permit sodium administration as early as the first day of life in very preterm infants (<28 weeks), with doses of up to 3 mEq/kg/day, particularly when parenteral nutrition is the primary fluid source [21].

A large Swedish observational study involving extremely preterm infants (EXPRESS cohort, n = 707) revealed that while 50% experienced hypernatremia during the first week, 79% developed hyponatremia during the second week [19]. Importantly, sodium supply and not fluid volume was identified as the primary determinant of plasma sodium levels. Their data emphasize the biphasic pattern in extremely preterm neonates: hypernatremia in the early days followed by hyponatremia unless sodium is adequately supplemented. Our findings support this trend, however, hyponatremia occurred earlier and more frequently in our cohort, likely due to differences in gestational age range and sodium initiation practices. This study also found that from Day 4 onwards, sodium supply was the sole predictor of hyponatremia (OR = 0.85, p<0.001), highlighting the importance of timely and adequate sodium supplementation. These findings corroborate our protocol of initiating sodium at 48 hours, though the persistence of hyponatremia on Day 3 in our study suggests that an even earlier supplementation may be warranted in selected neonates.

Another randomized clinical trial by Milani et al. investigated two IV fluid regimens in term neonates with sepsis: half-saline (7.7 mEq/kg/day) versus standard hypotonic fluid (3 mEq/kg/day). The half-saline group had significantly higher serum sodium at 24 and 48 hours and a lower incidence of hyponatremia, without an increase in hypernatremia or adverse effects [25]. These results suggest that even in term neonates, increasing sodium concentration in maintenance fluids can mitigate iatrogenic hyponatremia. Although our cohort included preterm neonates without confirmed sepsis, the implication for early sodium-enriched fluid strategies is relevant.

Together, these studies suggest that sodium supplementation plays a far more critical role than fluid volume in preventing both hypo- and hypernatremia. While concerns about hypernatremia exist, especially when sodium is introduced early, most of the studies did not find a clinically significant hypernatremia with modest sodium dosing. In conclusion, our results are similar to the accumulating evidence supporting the early and individualized use of sodium supplementation in preterm neonates. Judicious adjustment of sodium in maintenance fluids, particularly by Day 2 or 3 of life, may help prevent early hyponatremia without increasing the risk of hypernatremia, especially when guided by clinical and laboratory monitoring.

In our study, we observed a strong correlation between sodium values measured by BGA and laboratory autoanalyzer, with minor differences remaining within clinically acceptable limits. This aligns with the findings of Triplett et al., who reported a small but statistically significant mean difference of 1.49 mmol/L between BGA and lab results, concluding that both methods agreed well under most clinical conditions in ICU patients [15]. Zamanabadi et al. found higher BGA sodium values in adult ICU patients, especially in acidic pH states, supporting the need for cautious interpretation [12]. Another prospective study comparing 195 paired samples found no significant differences in sodium, potassium, and chloride levels between arterial blood gas and automated biochemistry analyzers. Strong correlation was observed, indicating that arterial blood gas results are reliable for critical care decision-making in electrolyte management [13]. The strong correlation between sodium values obtained from lab autoanalyzers and BGA suggests that these methods may be used interchangeably in clinical practice and can help minimize blood loss, procedural pain, and the risk of iatrogenic anemia in newborns, promoting safer and more efficient care.

However, several studies have highlighted notable discrepancies. Kim et al., in a large cohort of 450 preterm neonates, found a mean sodium difference of 4.2 mmol/L between arterial BGA and autoanalyzer, with BGA values significantly lower and a 51.9% hyponatremia detection rate versus 14.0% by lab values [14]. Another study by Kim and Kim found over 70% of very low birth weight infants had a sodium gap >4 mmol/L between the two methods, primarily influenced by low protein levels and greater physiological weight loss [5].

Doddamani et al. demonstrated that venous sodium levels were significantly higher than arterial values in neonates (135.94 ± 7.12 vs. 132.31 ± 11.31 mmol/L), attributing the difference to heparin interference and dilution in arterial samples, and potential preanalytical factors like hemolysis or ion binding [26]. Most studies using arterial BGA show lower sodium values compared to lab autoanalyzers. Since our study used venous samples for BGA, the sodium readings were naturally closer to laboratory values, minimizing bias and aligning with the evidence from the above study.

Limitations

This study has several limitations that should be acknowledged. First, the relatively small sample size of 30 preterm neonates limits the statistical power and generalizability of the findings. Larger multicentric studies would provide a more comprehensive understanding of the incidence of hyponatremia and enhance the validity of comparisons between different sodium measurement methods. Second, being a single-center study conducted in a tertiary care NICU in North India, the results may not be directly applicable to settings with different clinical protocols, levels of care, or patient demographics. Third, the follow-up period was restricted to the first week of life, potentially missing cases of late-onset hyponatremia that often develop beyond this timeframe in preterm infants. Additionally, although efforts were made to standardize sample collection and processing, pre-analytical variables such as sample handling time, heparin interference in blood gas syringes, and hemolysis could have influenced sodium values. The study also excluded neonates with congenital anomalies or those requiring surgical intervention, which may have led to an underestimation of electrolyte disturbances in a broader neonatal population. Lastly, factors known to affect sodium measurement by indirect ISE, such as serum protein and glucose levels, were not adjusted for, which could have introduced additional bias in the comparison of BGA and laboratory values.

## Conclusions

This prospective study highlights that hyponatremia is a frequent occurrence in preterm neonates receiving intravenous fluids, particularly during the early days of life. The incidence peaked around Day 5, with a notable decline by Day 7, likely influenced by timely sodium supplementation initiated at 48 hours of life. Despite this, a significant proportion of neonates remained hyponatremic on Day 3, suggesting a potential role for earlier sodium administration in selected high-risk infants.

Additionally, our findings demonstrate a strong correlation between sodium values measured using BGA and standard laboratory autoanalyzers. The differences between the two methods were generally within clinically acceptable limits, indicating that BGA can serve as a reliable tool for point-of-care electrolyte monitoring in the NICU when venous samples are used.

Overall, the study reinforces the importance of vigilant sodium monitoring and individualized fluid management in preterm neonates. Future research with larger, multi-center cohorts and extended follow-up is warranted to further refine sodium supplementation protocols and validate the accuracy of BGA in diverse neonatal populations.
